# Variations in the provision and cost of oral healthcare in 11 European countries: a case study

**DOI:** 10.1111/idj.12437

**Published:** 2018-09-19

**Authors:** Kenneth A. Eaton, Martin Ramsdale, Heather Leggett, Julia Csikar, Karen Vinall, Helen Whelton, Gail Douglas

**Affiliations:** ^1^ School of Dentistry University of Leeds Leeds UK

**Keywords:** Dental treatment, oral health, costs, Europe

## Abstract

**Aim:**

To compare the provision and costs at the point of delivery of dental treatments in a sample of European Union (EU) Member States.

**Materials and methods:**

A questionnaire with open‐ended questions was sent to oral health policy‐makers in Denmark, England, France, Germany, Hungary, Ireland, Italy, the Netherlands, Poland, Romania, Scotland and Spain. They were asked to answer questions on the probable costs and provision of treatment in their country for a vignette presented as a pre‐defined case.

**Results:**

All respondents returned answers to all questions. Wide variations were reported in: who would deliver care, cost of items of care and total cost. For example, in France, only a dentist would provide the treatment. In Denmark, England, Germany, Ireland, Italy, the Netherlands and Scotland, it was likely that the treatment would be provided by a combination of dentist, dental hygienist and dental nurse. Fees ranged from €72 in England (if treated within the NHS) to €603 in Denmark. In Italy, Spain and for most patients in Romania, all treatment costs were paid by the patient. In the other nine countries, some subsidy from public funds was available. In terms of percentage of per capita Gross National Income, the cost to the patient ranged from 0.12% in France to 1.57% in Spain.

**Conclusions:**

It was apparent that there are wide variations between EU Member States in the manner in which oral healthcare is delivered, its cost and the extent to which the cost of treatment is subsidised from state funds or through private insurance.

## Introduction

Six systems have been described for the provision of oral healthcare in Europe^1^. They have been termed Nordic, Bismarkian, Beveridgian, Southern European, Eastern European and Hybrid (publicly funded [free] oral healthcare for some or all children but largely private provision for adults). It is clear that there are wide variations between countries across Europe in the manner in which clinical oral health services are delivered to patients^1^, ^2^. Consequently, the costs of delivery of care vary greatly between countries. In a study of nine European Union (EU) Member States, England was found to be the most costly system for the delivery of dental care^3^. The study used a restricted vignette about the costs of placing a single amalgam restoration in a child, it was estimated that in 2005 the mean cost of such treatment across Europe ranged from €8 to €156. However, these costs were not those borne by the patients (or a third party) but were the actual costs of providing the care, i.e. those associated with diagnostic procedures, labour, materials, drugs and overheads. A more recent study published in 2015 compared fees in seven European countries (Denmark, France, Germany, Great Britain, Hungary, the Netherlands and Switzerland)^4^. It reported fees paid to dentists in terms of purchasing power parities, and overall found that they were lowest in France, Great Britain and Hungary^4^. There is very little published about the variations between countries from the patients’ perspective and/or knowledge of how healthcare is delivered in countries other than their own. There is a lack of understanding regarding which professionals deliver oral healthcare to patients, what types of treatments are most likely to be offered and who pays for the point of delivery costs, or price, of dental treatment. Information on these considerations will benefit those who seek oral healthcare when travelling to countries, other than their own, and healthcare planners and policy‐makers. The aim of this study was therefore to compare the provision and point of delivery costs (price) of dental treatments in a general dental practice in a sample of EU Member States using a well‐defined case description of a patient presenting for care.

## Materials and methods

**Figure 1:**
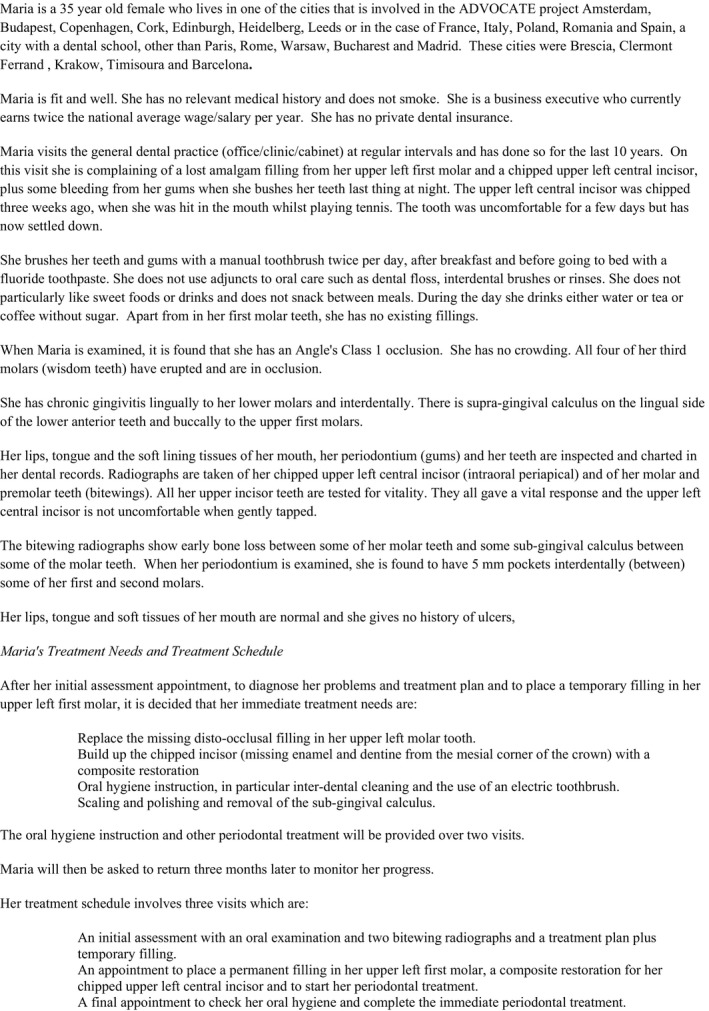
The vignette.

A scenario, or vignette (*Figure* 1), was developed for consideration by representatives of 11 EU Member States ensuring coverage of each of the six possible systems of oral healthcare provision^1^. The vignette detailed the case of a patient called Maria who required an examination, two small intraoral radiographs, two fillings and some relatively minor periodontal treatment. This vignette was used to explore how she would be treated in different Member States of the EU.

### Vignette design

The vignette was designed by the first author, and was reviewed by dental clinicians and administrators, prior to piloting in two countries (Ireland and UK). The reviewers were asked to check the vignette for clarity and relevance, and to suggest revisions if they considered them to be necessary. The vignette describes a fictitious patient and her treatment needs. The vignette was deliberately detailed so that those who reported how the patient (Maria) would be treated in their countries had a clear picture of the treatment that she required, her dental, medical and social history, and her ability to pay for treatment (*Figure* 1).

### Sample

Eleven EU Member States were selected to be included in the study to represent the full range of oral healthcare systems: Nordic – Denmark; Bismarkian – France and Germany; Beveridgian – UK (England and Scotland); Southern European – Italy and Spain; Eastern European – Hungary, Poland and Romania; and Hybrid – Ireland and the Netherlands. The total population of these countries combined is about 414 million people, just over 80% of that of the EU. Although England and Scotland are both part of one EU Member State, the payment system for oral health differs between these countries, so for the purposes of this study they were therefore treated as separate countries.For each of these countries key stakeholders were identified by the first author as they had current and detailed knowledge of the provision of oral healthcare in their country. The respondents included specialists in Dental Public Health, or senior administrators and current or past national Chief Dental Officers. Seven of the 12 respondents have recently published papers describing the systems for the provision of oral healthcare in France^5^, Germany^6^, Ireland^7^, Italy^8^, Poland^9^, Romania^10^ and Spain^11^, and in so doing had readily available information and data to help them answer many of the questions on the vignette. All had previously published in English language journals, and did not require the vignette or questions translating into their own language. In countries such as England, France and Scotland, where there are fixed fees within a national oral healthcare system, respondents were able to report these fees direct from published lists of fees. In other countries they contacted a range of general dentists, who owned their own office/cabinets/clinics to obtain average fees for the country concerned.

The Dental Research Ethics Committee of the University of Leeds gave ethical approval for the study (reference 051115/HL/182). The study was conducted in full accordance with the World Medical Association Declaration of Helsinki. All those who reported for each of the countries confirmed their consent to do so and willingness to be named in the acknowledgements section of this paper in writing (by email).

### How would Maria be treated in general dental practice in each of the countries?

Having read the vignette (*Figure* 1), the respondents were asked to answer the following questions with detailed comments and return them by email.

- Parts of the treatment plan will be provided by dentists, dental hygienists, dental nurses?
  ° Taking the radiographs
  ° Carrying out the assessment and treatment planning
  ° Placing the filling and the composite restoration at the upper central incisor
  ° Will the missing amalgam filling in the upper left molar be replaced by another amalgam or by a composite filling and why?
  ° Giving oral hygiene advice
  ° Scaling and polishing, and the removal of sub‐gingival calculus
- Will dental nurses be assisting dentists and dental hygienists when they provide the treatment?
- How many other dentists will be working in the dental practice concerned?
- How likely is it that the dentists will be male or female?
- How likely is it that their original qualification was from a country other than the one in which they are now working?
- How much will the treatment cost?
- Will some of the costs be paid for by public or private health insurance?
- If so how much?
- If all or some of the treatment cost is not covered by national health insurance, how much will Maria have to pay for each part of the treatment and in total?

Data that were included in the Council of European Chief Dental Officers (www.cecdo.org) database were compared with those provided by respondents. Other data and information provided by the respondents were not checked against the CECDO database. However, all the authors are currently working on a 4‐year European Commission‐funded project (ADVOCATE), which involves the use of big data to improve the provision of oral healthcare. As part of this project they have reviewed systems for oral healthcare in several EU countries. The first author has advised the CECDO for over 25 years, and has published widely on the topic of oral healthcare in Europe. The authors critically appraised the responses and, where there were apparent anomalies, the respondents were contacted to resolve them.

## Results

Over a 4‐month period in 2016, all 12 respondents (100% response) submitted answers to all the questions. If some answers were unclear they were contacted to provide clarification. The data were therefore current for 2016.

### Which parts of the treatment plan will be provided by dentists, dental hygienists, dental nurses?

In all 12 countries, dentists were reported as being able to perform all of the treatment within the treatment plan (*Table* 1). In 11 countries, dental hygienists could perform some of the treatment, the exception being France where dental hygienists are not trained and this role does not exist within the dental workforce. In 11 other countries, if the practice employed a dental hygienist, they could give oral hygiene, scale and polish, and remove sub‐gingival calculus. In eight countries (Denmark, England, Hungary, Ireland, Italy, the Netherlands, Scotland and Spain), if they had received training they could take the radiographs; in three (Denmark, Ireland and the Netherlands), if requested to do so by the dentist, place a temporary filling; and in one (Denmark) place a filling. In Denmark and the Netherlands it was possible for the dental hygienist to carry out an assessment and make a treatment plan. The role of dental nurses also varied widely between the 12 countries. In none of the countries were dental nurses allowed to perform an assessment and make a treatment plan. In nine of the countries, if dental nurses had received additional training and there was a prescription from the dentists they could take radiographs. In Germany it was reported that if dental nurses had undertaken a full course of training in extended duties they could also perform supra‐gingival scaling and polishing. In Denmark, also after further training, it was reported that dental nurses could place a filling under the supervision of a dentist, and perform supra‐gingival scaling and polishing. In seven of the countries (Denmark, England, Germany, Ireland, the Netherlands, Romania, Scotland) after suitable training, dental nurses were reported as being allowed to give oral hygiene instruction. In France, it was reported that dental nurses were not allowed to perform any of the treatment within the treatment plan (*Table* 1).

**Table 1:** Who may perform the treatment

Country	Role	Exam and treatment planning	Radiographs	Temporary filling	Restoration with amalgam	Restoration with composite	Oral Health Instruction	Scaling
Denmark	Dentist	Yes	Yes	Yes	No	Yes	Yes	Yes
	Dental hygienist	Yes^a^	Yes	Yes	No	Yes^b^	Yes	Yes
	Dental nurse	No	Yes^c^^,^ d	Yes	No	Yes^b^	Yes^,^ c ^,^ d	Yes^c^^,^ d
England	Dentist	Yes	Yes	Yes	Yes^e^	Yes	Yes	Yes
	Dental hygienist	No	Yes	No	No	No	Yes	Yes
	Dental nurse	No	Yes^c^	No	No	No	Yes^c^	No
France	Dentist	Yes	Yes	Yes	Yes	Yes	Yes	Yes
	Dental hygienist^f^	No	No	No	No	No	No	No
	Dental nurse	No	No	No	No	No	No	No
Germany	Dentist	Yes	Yes	Yes	Yes^g^	Yes^h^	Yes	Yes
	Dental hygienist	No	Yes^c^^,^ d	No	No	No	Yes	Yes^d^
	Dental nurse	No	Yes^c^^,^ d	No	No	No	Yes	Yes^c^
Hungary	Dentist	Yes	Yes	Yes	Yes	Yes^i^	Yes	Yes
	Dental hygienist	No	Yes	No	No	No	Yes	Yes
	Dental nurse	No	Yes^c^	No	No	No	No	No
Ireland	Dentist	Yes	Yes	Yes	Yes	Yes	Yes	Yes
	Dental hygienist	No	Yes^j^	Yes	No	No	Yes	Yes
	Dental nurse	No	Yes^c^^,^ j	No	No	No	Yes^c^	No
Italy	Dentist	Yes	Yes	Yes	Yes	Yes	Yes	Yes
	Dental hygienist	No	Yes^d^	No	No	No	Yes	Yes
	Dental nurse	No	Yes^d^	No	No	No	No	No
Netherlands	Dentist	Yes	Yes	Yes	Yes	Yes	Yes	Yes
	Dental hygienist	Yes (possibly)	Yes	Yes	No	No	Yes	Yes
	Dental nurse	No	Yes^j^	No	No	No	Yes	No
Poland	Dentist	Yes	Yes	Yes	Yes^k^	Yes	Yes	Yes
	Dental hygienist	No	No	No	No	No	No	Yes^d^
	Dental nurse	No	No	No	No	No	No	No
Romania	Dentist	Yes	Yes	Yes	No	Yes	Yes	Yes
	Dental hygienist	No	No	No	No	No	Yes	Yes^j^
	Dental nurse	No	Yes^g^	No	No	No	Yes	Yes
Scotland	Dentist	Yes	Yes	Yes	Yes^e^	Yes	Yes	Yes
	Dental hygienist	No	Yes^c^	No	No	No	Yes	Yes
	Dental nurse	No	Yes^c^	No	No	No	Yes^c^	No
Spain	Dentist	Yes	Yes	Unlikely to be placed	Yes	Yes	Yes	Yes
	Dental hygienist	No	Yes^c^	No	No	No	Yes	Yes^j^
	Dental nurse	No	Yes^c^	No	No		No	No

a Straightforward cases only.

b Since 2007, specific conditions.

c Following additional training.

d Supervised by a dentist.

e In molars only amalgam paid for by the NHS.

f Not trained in France.

g Amalgam paid for by public insurance.

h Additional fee depending on the size and localisation of the filling.

i Amalgam usually replaced with composite.

j When prescribed by a dentist.

k Only amalgam paid for by the state.

By general nurses in large dental clinics or specialised centres.

### Will dental nurses be assisting dentists and dental hygienists when they provide treatment?

In nine of the countries, all except France, Poland and Romania, it was reported that the dentist would be assisted by a full‐time dental nurse. In two of the countries (France and Romania), dental nurses also act as receptionists and may not be present at the chair‐side all the time to assist the dentist. In the 11 countries, where dental hygienists work, it was reported that with one exception (Spain) they either always or usually work without a dental nurse to assist them.

### How many other dentists will be working in the dental practice concerned?

Seven countries (France, Hungary, Ireland, Italy, Poland, Romania, Spain) reported that usually only one dentist works full‐time in a practice, although they may employ visiting orthodontists or implant specialists. In the North‐Western, European countries (Denmark, England, Germany, the Netherlands and Scotland), it was reported that only a minority of dentists now work as the only general dentist in a practice (office/clinic).

### How likely is it that the dentist will be male or female?

The male:female ratio varied from 25%:75% (Poland) to 66%:34% (Italy). In five of the countries it was reported that there were more female than male dentists. The respondent from Germany reported that in 2015 the intake of students to German dental schools was 30% male and 70% female. The trend for more female than male dental students appears common in Northern, Western and Southern European countries.

### How likely is it that their (the dentist's) qualification was from a country other than the one in which they are now working?

In terms of the percentage of registered dentists who qualified in another country, the UK had the highest with 28% in England and Scotland. In the UK, as a whole at the end of 2016, out of 41,482 registered dentists, 11,612 had graduated as dentists in another country^12^. For Ireland 25% and Spain 16% it was also reported that Maria might well be treated by a non‐national dentist. Other than in Denmark, where it was reported that 15% of dentists had qualified in another country, in the remaining seven countries the percentage of such dentists was 10% or lower (*Table* 2).

**Table 2:** Dental workforce characteristics – France, Italy, Spain, Romania and the ADVOCATE Project Countries as at 1 April 2016

Country	Working dentist numbers	Number of registered dental hygienists	Number of dentists/practice	Number of dental nurses	Number of hygienists/practice	Male:female ratio of dentists %	Practice owner private/corporate/publically funded	Dentists that graduated from another country
Denmark	4,800	1,751	Generally two or more	At least one per dentist	The majority of practices employ a dental hygienist	26/74	Often ‘large dental clinics’	480 10%
England	41,482 (UK total)	6,385 (UK)	In over 80% of practices two or more	At least one per dentist	The majority of practices employ a dental hygienist, often part‐time	54/46	60% two or more practice owners (co‐owners) < 20% owned by single dentist < 20% owned by companies	11,612 (UK) 28%
France	41,795	0	Generally only one	50% work without a full‐time dental nurse	No dental hygienists are registered to work in France	58/42	Generally owned by the dentists who works there	1,513 4%
Germany	70,740	600 (estimate)	1–3 tendency for larger dental clinics with several dentists and specially trained dental chairside assistants to become the norm	Often two per dentist, many of whom have further training	There are fewer than 600 dental hygienists in Germany, but over 13,000 dental nurses with additional training who carry out supra‐gingival scaling	Approaching 50:50 in practice 30:70 student intake	Generally owned by the dentist or dentists who work(s) there	In 2014 there were 2,163 dentists who had qualified in a country other than Germany, i.e. 3%
Hungary	5,420	2,077		In general, most Hungarian dentists work with a dental nurse	In 2010, there were 2,077 dental hygienists, but many are working as dental nurses	38/62 (2015)	Owned by one dentist or an investor	In 2017, about 1% of active Hungarian dentists qualified from another country, nearly all had a Romanian qualification
Ireland	2,828	487	One (majority)–two or more is likely in future (average 2.8 in multi‐surgery practice)	Generally one per dentist	In 2016, there were 487 dental hygienists in Ireland	53/47	Mixture of corporate and non‐corporate ownership	683 25%
Italy	59,324	4,000	Usually one but with visiting specialists	One per dentist		66/34	One practice owner (dentist) – 80% of all practices 10% company run – ‘dental services’	1,172 2%
Netherlands	8,827	2,850	Usually more than 1 (1.8 is the average)	Two per dentist	In 2012 there were 2,850 dental hygienists, some of whom owned their own practices	60/40		799 9%
Poland	40,116	2,260	Usually one, but sometimes with visiting specialists	In the public dental service in 2014 : 13,056 dentists and only 5,288 dental nurses	Most likely 0	25/75	Majority owned by a single practitioner (77%, 2014) or private healthcare clinics	600 1.5%
Romania	15,396	100	Generally 1, but increasing numbers of dental companies with multiple dentists	As there are 6,000 dental nurses it appears that many dentists work without dental nurses	Most likely 0	32/68	Owned by a single practitioner	485 3%
Scotland	414,820 (UK)	6,385 (UK)	In the majority of practices two or more	At least one per dentist	The majority of practices employ a dental hygienist, often part‐time	54/56	Two practice owners (co‐owners) < 20% owned by single dentist < 20% owned by companies	11,612 (UK) 28%
Spain	34,200	13,200	Usually one but sometimes with visiting specialists	At least one per dentist	The majority of practices employ a dental hygienist, often part‐time	48/52	Generally owned by one private dentist (most cases but decreasing), increasing number of company operated (with 6–10 dental units per practice)	5,879 16%

Data sources: answers from respondents in this current study plus Council of European Chief Dental Officers database www.cecdo.org.

### How much will all the treatment cost?

This question asked for the overall cost, i.e. the total fee that the dentists will receive. Assuming that Maria received a composite filling for her upper molar, the total reported cost of treatment ranged from €158 in France to €603 in Denmark. In Spain it was €510, in the Netherlands €505, in Germany €448 and in Ireland €458. However, if Maria accepted an amalgam filling in her upper molar in both England and Scotland, all her treatment could be performed within the NHS. As a Band 2 treatment in England the overall cost would have been €72 and in Scotland, which still operates a fee for item system, it would have been €124 (*Table* 3).

**Table 3:** Expected total cost of treatment for scenario‐based items in each of the countries analysed together with cost incurred by the patient and by the state

Country	Total fee in €	Cost paid by the patient in €	% of overall treatment cost covered by the state	*GNI per capita PPP in € in 2016	Cost of treatment to patient as percentage of per capita GNI
Denmark	603	465 paid by patient of which 138 paid by public (state) health insurance scheme	23%	45,027	1.03%
England £1 = €1.20	€72 If all treatment covered by band 2 NHS charge, if private elements incorporated then up to €323	NHS 85.3% paid by a non‐exempt charges patient (€61 for a band 2 course of treatment, same charge in England and Wales at all NHS practices)	Dependent on UDA/contract value, if UDA = €24 then band 2 (3 UDAs = €72) therefore state would contribute 14.7%	UK 38,189	0.16% (NHS only) 0.82% (private fee)
France	158	47, which is reimbursed if patient has private insurance (95% population have complementary insurance) they would pay this additional amount so patient overall charge would be 0)	70% of cost (111) reimbursed by public health insurance system (all items discussed in this scenario are regulated by this system, of which 98% of dentists have a contract)	38,114	0.12% (without private insurance)
Germany	448	200	248	45,451	0.44%
Hungary 1HUF = 0.0032 EURO	250 ± 100 for treatment in private dental office, free if treated in either a state‐owned or contracted dental office	100% if private, 0% if regional practice has contract with Health Insurance Office of Hungary	100% if regional practice has contract with Health Insurance Office of Hungary	23,242	1.09% (If costs entirely private)
Ireland	205 without additional periodontal treatment, 425 including two quadrants of periodontal scaling plus €33 paid by social insurance = total of €458	100% (45.8% of populous have private health insurance, which will reimburse €25 per appointment up to a maximum of 10 appointment per policy), tax relief at 20% and private dental insurance may reduce costs further	€33 paid by social insurance	43,708	1.04%
Italy	380	100% (0% if patient has insurance but only 10% patients have this)	0%	33,763	1.13%
the Netherlands	505	100%, but if voluntary supplemental insurance reimbursement varies between 75% and 100% up to a maximum that varies between 100 and 1,750	0%	45,582	1.10%
Poland	Approximately 260	100% if private, though 15% of population receive state‐funded dental treatment (conservative dentistry, preventative dentistry and oral surgery)	Composite fillings not provided by the state, amalgam paid for by state insurance system	23,921	1.09% (private treatment)
Romania	210	40% of fee (if dentist has a contract with the National Health Insurance House – minority)	If dentist has a contract with the National Health Insurance House – minority of practices, if so 60% paid for by the state but fixed at €300 paid to general dentists and 400 to specialist dentists	19,683	1.07%
Scotland £1 = €1.20	NHS only‐ €123.60 (patient charge €99), private – periodontal and composite restoration in molar (same as England) + NHS charge would be = €251 private composite molar tooth and periodontal care, NHS radiographs and incisal composite (€36) = €287	80%	20%	UK 38,189	0.26% (if all NHS) 0.73% (if private for periodontal care and composite filling in upper molar)
Spain	510	100 (< 1% population covered by private insurance)	0 (only extractions covered by Spanish NHS equivalent, salaried dentists perform this)	32,482	1.57%

### Will some of the costs be paid for by public or private health insurance? If so, how much?

In some of the 12 countries Maria could receive some subsidy towards the total fee. The extent of the subsidy ranged from 0% from public funds in Italy, the Netherlands and Spain to 70% from the state health insurance scheme, plus the remaining 30% from a complementary private insurance scheme (i.e. 100% in France) and also 100% in Hungary, if Maria had been treated by a dentist who had a contract with the Health Insurance Office of Hungary.

### If all or some of the treatment cost is not covered by national health insurance, how much will Maria have to pay for each part of the treatment and in total?

The costs of the individual elements within the overall treatment varied widely, and in Italy it was reported that there was no fee for the radiographs or the temporary filling. Where private fees were charged, such as for the composite filling in the upper molar, some respondents quoted a range of fees that individual dentists would charge. In these cases the mean was taken as the likely fee. The details of how the fees for each element of the treatment plan varied from country to country will be reported in a second paper.

As far as the total personal payment that Maria made is concerned, this ranged from €47 in France to €510 in Spain (*Table* 3). In Spain, only 1% of the population is covered by private oral health insurance. In the Netherlands, as previously mentioned, patients with supplementary private insurance can claim reimbursement.

As the purchasing power of the Euro varies from country to country, the cost she paid in each country has been compared with the average per capita Gross National Income for 2015 (GNI) to facilitate the comparable relative cost to Maria. The average GNI per capita ranged from €19,683 in Romania to €45,582 in the Netherlands. It was over €38,000 in Denmark, England, France, Germany, Ireland, the Netherlands and Scotland, and < €23,000 in Hungary, Poland and Romania. Using this comparator, the cost to Maria would have been lowest in France at 0.12% of GNI and in England at 0.16% of GNI (if the treatment had been performed within the NHS), and highest in Spain at 1.57% of the mean GNI and in Romania at 1.51% of GNI (*Table* 3). However, as described in the vignette, as Maria's annual income was equivalent to twice the national average wage/salary in the more affluent countries, she would have had to pay a lower percentage of her annual income than indicated in the previous sentence.

## Discussion

Nearly 20 years ago, one study^13^ concluded that despite the wealth of international comparative work on healthcare and medicine more generally, the analysis of oral healthcare across international borders is relatively neglected. This statement is particularly true for cost comparisons. The studies that have been performed have invariably investigated the total spend on oral healthcare in countries, and the proportions funded by public and private sources^13^, ^14^, ^15^, ^16^ or on total expenditure on oral health in European countries^17^, ^18^.

A review of the literature found only two previous studies that had sought to compare the cost of specific items of dental treatment across European countries^3^. The first, published in 2008, was carried out by a team of health economists and investigated the actual cost (including dentists’ salaries, materials and other overheads) of providing a restoration (filling) in a molar tooth in a 12‐year‐old child in nine European countries, rather than the price charged for the treatment. There was no detailed clinical specification, such as size of the restoration, material to be used or the type of clinic in which the procedure would take place. As a result, the cost for providing the filling in one of the countries involved (England) was far higher than in the others because it related to the costs incurred in a special needs clinic rather than a general dental practice. In another country, the filling was placed in a state clinic and in others in general dental practices. These issues creating incomparability were addressed in the current study by presenting the participants with a detailed medical, dental and social history and clearly defined treatment needs. The current study also considered the price of treatment at the point of delivery, i.e. the monetary value that the dental team would receive from the patient/third party. The second study^4^ was carried out for the Institute der Deutschen Zahnärtze (IDZ) and was published in 2015. It compared dental fees (in Denmark, France, Germany, Great Britain, Hungary, the Netherlands and Switzerland) using purchasing power parity as a basis for the comparison of the prices for 11 items of treatment. Only three of these items of treatment (examination and consultation, two surface fillings, and sub‐gingival curettage) from the 2015 study^4^ were included in the vignette used in the current study. The IDZ study^4^ found that prices for these three items of treatment were lowest in France, Great Britain and Hungary, confirming the results of the current study for these three items of treatment.

The costs reported do not include travel costs and the cost of time off work for patients. They also apply purely to general dental practices and not to public clinics or hospitals, or other locations where oral healthcare is provided. A further possible limitation is the accuracy of the reported costs. Where the treatment is provided in countries with a fixed scale of fees for oral healthcare they should be accurate, for example in England, France, Germany and Scotland. However, even in the public systems in Germany (Krankenkasse) and England (NHS), there are variations. In Germany, fees are based on a points system and the value of a point varies from region to region. In England, within the NHS the cost to the patient is consistent; however, the value of Units of Dental Activity (UDAs), which are used to remunerate general dentists, vary. In countries where the patients are not treated within a state‐funded system, or in a private insurance system with a fixed tariff of fees, the resulting private fees reported in this study (paid direct from patient to dentists without insurance cover) were invariably an estimate and should be viewed as such. Nevertheless, because respondents were asked to report ‘average fees’ for general dental practices not located in capital cities, where the costs may have been higher and sought this information from a range of general dentists, it may be that the ‘private fees’ reported are typical for the countries concerned. It would be most unwise to assume that they are exact. Nevertheless, they do give an overall picture of oral healthcare fee relativity in the 12 countries, and for the countries included and items of treatment concerned. Fees for the care and treatment of children or for the provision of treatment involving laboratory costs were not included in the vignette. If they had been it is possible that a different picture may have emerged.

A wide variation in who (dentists, dental hygienist or dental nurse) provides oral healthcare in the 12 countries was apparent and merits further investigation. For example, why has the concept of team dentistry not been accepted in France, and to what extent does the lack of dental nurses influence the quality of care in that country?

In summary, the results of this study reflect the wide variation in cost of oral healthcare between the countries and the way in which it is provided. This was perhaps unsurprising as there is no over‐riding EU policy on systems for the provision of healthcare in its Member States. Apart from costs, the variation in the manner in which oral healthcare is provided in the countries that took part in this study has been highlighted in the Results section of this paper. It is clear that there are wide variations between the countries in who delivers oral healthcare, how much it costs and who pays. It seems probable that the general populations of European countries are unaware of these variations and, in general, may only know answers to these questions for their own countries.

## Conclusions

From the data gathered in this study, it is apparent that there are wide variations between EU Member States in the manner in which oral healthcare is delivered, its cost and the extent to which the cost of treatment is subsidised either from state or private funds.

### Funding

All the authors are involved in ADVOCATE, a Horizon 2020 programme project (number 193321) funded by the European Commission, and some or all of their salaries are paid from this source of funding.

### Conflict of interest

None other than as a possible result of the source of funding.

### Author contribution

Kenneth Eaton planned the study, wrote the scenario and the first draft of the paper. He subsequently edited further drafts and the final version of the paper. Martin Ramsdale analysed the data and produced the tables. Heather Leggett contributed to creation of the scenario and to the writing of the paper. Julia Csikar contributed to creation of the scenario and to the writing of the paper. Gail Douglas planned the study, contributed to the scenario and contributed to the writing of the paper. Karen Vinall contributed to the writing of the paper. Helen Whelton contributed to creation of the scenario and to the writing of the paper.
